# Stimulus Selection Based on Gender Ratios: Gender Ratios are Indicative of Both Stereotypical and Conceptual Gender

**DOI:** 10.1177/00332941241253582

**Published:** 2024-05-14

**Authors:** Jonathan D. Kim, Ute Gabriel, Pascal Gygax, Anna Siyanova- Chanturia

**Affiliations:** Department of Psychology, 8018 Norwegian University of Science and Technology, Trondheim, Norway; Department of Psychology, 30475 University of Fribourg, Fribourg, Switzerland; School of Linguistics and Applied Language Studies, Te Herenga Waka - Victoria University of Wellington, Wellington, New Zealand

**Keywords:** Gender ratio, gender stereotypicality, conceptual gender, attributes, components

## Abstract

This paper explores how the use of gender ratios to inform stimulus selection affects the activation of gendered social information. It investigates if stimuli selected this way can activate gender stereotype knowledge and/or conceptual gender knowledge. This was tested through attribute naming (Study 1) and rating (Study 2) tasks, with component and regression analysis allowing for examination of the nature of gender ratios at both attribute and component levels. The results provide rich information about the nature of gender ratio information as a means of stimulus selection, and in doing so support both conceptualisations as long as researchers acknowledge their overlap. The results also indicate that these roles elicited both positive/prescriptive (i.e., the role is appropriate for a given gender) and negative/proscriptive beliefs (i.e., the role is *not* appropriate for a given gender). These findings hold important implications for future research using gender ratios.

## Introduction

Gender is so strongly linked with humanness that, when an individual is mentioned in text or speech, we mentally assign a gender to them – even when gender is neither referenced nor relevant (Cacciari & Padovani, 2007; Carreiras et al., 1996; Irmen & Roßberg, 2004; Stahlberg & Sczesny, 2001). When gender is not explicitly indicated, this happens based on implicit information sources (Banaji & Hardin, 1996; Reynolds et al., 2006). A common source of implicit information is the gender ratio (i.e., the [real or assumed] comparative ratio of different genders) of any social and occupational group(s) the referenced individual is perceived as belonging to. Research in this area has heavily focused on binary gender distinctions (i.e., between female-dominated, balanced [i.e., 50/50 female to male], and male-dominated roles), with much less known about roles dominated by third-gender groups (non-binary, agender, etc.). Further, even within the gender binary, the exact way gender ratios guide perception is not a settled debate.

Gender ratios can broadly be divided into *real* gender ratios, which are based on actual gender distributions in a population, and perceived gender ratios, which are based on social perceptions of gender distributions. Research has found that real and perceived gender ratios are often very similar (e.g., Adachi, 2013). Gender ratios are utilised by researchers across numerous disciplines, such as linguistics, psychology and psycholinguistics, to perform various activities. These activities include guiding paradigm and stimulus material selection, providing structure for data analysis, and guiding the reporting of results. The popularity of gender ratios in research is widespread due to the perception that gender ratios are representative of broader, yet competing, factors. In this paper we focus on two of these conceptualisations; namely, gender ratio as a measure of gender stereotypicality (e.g., Carreiras et al., 1996; Glick et al., 1995; Gygax et al., 2016; Kennison & Trofe, 2003), and/or as a measure of conceptual gender (e.g., Gygax et al., 2016; Irmen, 2007; Kotek et al., 2021). Broadly, these conceptualisations assume that gendered information provided by gender ratios initially guides categorisation, and then either remains salient, affecting social perception (for gender stereotypicality), or stops being salient (for conceptual gender). These conceptualisations are complicated by two historic but untested assumptions. Firstly, the assumption that gender ratio information supports either conceptualisation. Secondly, the assumption that the conceptualisations largely or fully overlap. In this paper we seek to address these assumptions by examining the types of gendered information that stimuli selected based on gender ratio activate. We do so through a ground-up exploration of the attributes and components associated with different roles. Due to insufficient knowledge about the roles dominated by the third gender, we examined female-dominated, balanced, and male-dominated occupational roles. To position this paper, we discuss gender stereotypicality and conceptual gender as separate constructs, as well as the specific ways in which gender ratios can be conceptualised as a measure of both.

### Gender Stereotypes

Gender stereotypicality can be defined as the gender-based beliefs held about individuals and groups, including beliefs around social and occupational roles. These individual and group level beliefs are complementary (e.g., Eagly & Wood, 2016); for example, women are perceived as best suited in feminine stereotyped roles, particularly those who display feminine attributes (e.g., caring, helpful), while men are perceived as best suited in masculine stereotyped roles, particularly those who display masculine attributes (e.g., aggressive, competitive).

The conceptualisation of gender ratios as a measurement of gender stereotypicality draws upon two rationales. Firstly, social stereotypes are internalised at a young age, and individuals make decisions for their futures (e.g., which occupation[s] to work in as adults) based on gendered self-stereotyping (e.g., Eccles, 2011). Secondly, gender stereotypical behaviour, such as women working in female stereotyped occupations, is perceived to be more socially desirable than gender counter-stereotypical behaviour; i.e., women working in male stereotyped occupations (Prentice & Carranza, 2002), with counter-stereotypical behaviour resulting in social and/or economic penalties (Fiske et al., 1991; Harrison et al., 2012; Rudman & Glick, 1999; Shaw & Hoeber, 2003). As such, gender stereotypes are held to predominantly account for gender segregation in the workforce, as reflected in gender ratios. This conceptualisation has been supported by research such as that by Adachi (2013), who found that female-dominated occupations were seen as highly female stereotyped (and were therefore associated with female stereotyped attributes), while male-dominated occupations were seen as highly male stereotyped (and were therefore associated with male stereotyped attributes). For the sake of the argument in this paper, we take the conceptualisation of gender ratio as a measure of gender stereotypicality to its logical extreme, where there is no overlap in the gender ratio information associated with each of the gender stereotype categories (feminine, masculine, neutral).

### Conceptual Gender

Conceptual gender can be defined as the gender-based categorisation of objects or roles based on lexical semantics or stereotypical knowledge, in a manner that cannot *directly* be traced to linguistic or natural gender categories (Irmen, 2007; Sera et al., 1994). An example is the categorisation of natural objects (e.g., plants) as feminine and artificial objects (e.g., hammers) as masculine (e.g., Ortner, 1974; as cited in Sera et al., 1994). When it comes to conceptual gender categorisation related to humans and human activities, it *generally* follows the individual(s) gender identity, and/or the gender typically associated with the specific role (Vigliocco et al., 2005).

Some researchers suggest that, as gender stereotypes and conceptual gender both derive from generalised beliefs about what gender categories entail, both conceptualisations should fully or partially overlap (e.g., Gygax et al., 2016; Kotek et al., 2021). Indeed, Irmen (2007) references research examining gender stereotypicality as if it had examined conceptual gender. However, as conceptual gender categorisation can occur based on lexical semantics with or without stereotype activation, conceptual gender cannot be universally assumed to be in line with gender stereotypes. For example, lexically gendered terms such as *waiter* and *waitress* activate gendered representations based on lexical markers without *necessarily* activating stereotypes. Further, while the process of conceptual gender categorisation can still draw upon both lexical and stereotypical gendered expectations, it is purely a method of *classifying* referents into gendered groups (e.g., Ackerman, 2019). As such, categorisation can be initially driven by gender stereotypes (as suggested by Gygax et al., 2016), lexical semantics, or both. Following categorisation, this information is believed to be deactivated, and is potentially replaced with gender information associated more broadly with the category.

For the sake of the argument in this paper, the deactivation of gender information is taken to its logical extreme: conceptual gender is held to abandon all gendered expectations following categorisation. Under this assumption, conceptual gender categories are therefore simple categories. The concept of gender ratio as a measure of conceptual gender is based on the idea that these ratios permit classification based on the percentage of women and men in each role, thereby reducing the need for stereotypical information when classifying, hence simplifying the identified gender categories.

### The Present Study

Gender stereotypicality holds that gender ratios allow for the identification of complex categories (i.e., connected to, and therefore activating, gender stereotyped beliefs), while conceptual gender holds that gender ratios allow for the identification of simple categories (i.e., minimally connected – or even unconnected – to gender stereotyped beliefs). Due to the fundamental difference in these conceptualisations, care must be taken when assuming any level of overlap between them, and when choosing one or the other for guiding any part of the research process. We contend that, while the use of gender ratios for selecting experimental stimuli may not be directly affected by the conceptualisation used, the *interpreted* findings of a study cannot always be assumed to overlap, as any interpretation is grounded in the author’s conceptualisation of the true nature of gender ratios.

In this paper, we conduct research that examines the gendered beliefs activated by occupational roles that were selected based on their perceived gender ratios. The specific occupations chosen were selected based on the perceived gender ratios identified in Misersky et al.’s (2014) research, which has been used previously to guide stimulus material selection for research on both conceptual gender and gender stereotypicality (Adachi, 2013; Gygax et al., 2016; Irmen, 2007). These beliefs are explored using a revised version of Koivula’s (2001) paradigm for exploring gender stereotypes in sports. In this paradigm, gendered beliefs are examined through a spontaneous attribute naming task (Study 1) and through an attribute rating task (Study 2). The spontaneous attribute naming task is a ground-up approach permitting participants, as opposed to researchers or existing literature, to nominate the attributes. The attribute rating task, contrasting this, solicits a distinct group of participants to rate the importance of these attributes for one female-dominated, one balanced, and one male-dominated occupation each. The results of these studies allow for the examination of the comparative importance, and frequency of naming, for attributes and components relating to occupations with differing gender ratios. Through both spontaneous naming and rating tasks, the underlying beliefs associated with these roles are documented. The exploration of these findings will enable us to ascertain whether the beliefs activated by these roles align with gender stereotypicality or conceptual gender.

As the beliefs associated with roles based on their gender ratios have not been comprehensively explore before, this research is exploratory in nature. As such, we have no hypotheses about the nature of these beliefs. In relation to the extreme versions of the competing conceptualisations of gender ratio – as a means of selecting stimuli indicative of gender stereotypicality (all gender information remains relevant, attributes and components differently important for each gender category) or of conceptual gender (no gender information remains relevant, attributes and components equally important for all gender categories) – we expect that responses should either show clear distinctions between gender categories (in line with gender stereotypicality) or no distinctions between gender categories (in line with conceptual gender).

The studies’ results undergo frequency analysis, principal and rotated component analysis, and linear mixed-effects regression. Both attribute and a component (based on the component analysis) levels undergo frequency analysis and linear mixed-effects regression. The full analysis battery requires both studies’ outputs, therefore the methods sections for both studies are presented before the combined results section.

## Study 1

### Method

#### Participants

Thirty participants took part in this study (14 female, 13 male, 3 non-binary), of which 19 were university-level students. Participants were between 18 and 38 years old (*M* = 22.7, *SD* = 4.5), and were recruited via direct recruitment in and around university campuses in Wellington, New Zealand. All participants were self-reported native New Zealand English speakers. Participants were compensated with $5 NZD. This sample size matched that of Koivula (2001), who conducted research using a very similar paradigm, and was determined prior to data analysis. Ethical approval for this study was given by the Human Ethics Committee at Victoria University of Wellington.

#### Materials and Research Design

A two-step questionnaire was used to gather responses. All experimental elements were presented in English. Participants gave informed consent, answered questions on age, gender, and first language fluency, and stated whether they were currently enrolled university level students prior to experimental onset. Participants undertook this experiment through paper-based forms.

Participants were presented with lists of 36 predetermined occupations and were asked to select one female-dominated, one balanced, and one male-dominated occupation that they were the most familiar with, and one occupation that they were the least familiar with. The occupations list was composed of 36 occupational roles (12 female-dominated, 12 male-dominated, and 12 balanced; see Table 1) selected based on their perceived gender ratios, as reported by Misersky et al. (2014). The female- and male-dominated occupations were selected so that they were, as much as possible, significantly (and equally) different from the balanced (i.e., 50/50 women to men) ratio in opposite directions, while the balanced occupations were selected so that they were as close to an equal female to male ratio as possible. Further, occupations with explicit gender markings (e.g., ‘firemen’) were excluded from selection to ensure participants responses were based on implicit beliefs associated with occupations and not on explicit gendered beliefs associated with gender markings. Misersky et al. (2014) presented their results on a continuum based on the perceived percentage of women in the role, from 0 (0% women) to 1 (100% women). Based on this continuum, the female-dominated occupations used in this study had a perceived mean ratio of 78% women (*SD* = 4%), while the male-dominated occupations had a perceived mean ratio of 21% women (*SD* = 4%), and the balanced occupations had a perceived mean ratio of 50% women (*SD* = 3%). Occupations were presented in alphabetical order throughout the questionnaire.

**Table 1.** Perceived Gender Ratios for Occupational Role Nouns in English, as Determined From the Findings of Misersky et al. (2014).

Female-dominated	Score	Balanced	Score	Male-dominated	Score
Beauticians	.84	Artists	.51	Astronauts	.23
Birth Attendant	.75	Authors	.51	Boxers	.21
Dressmakers	.75	Entertainers	.46	Bricklayers	.16
Florists	.77	Environmentalists	.54	Bus drivers	.25
Fortune tellers	.75	Jewellers	.49	Carpenters	.25
Housekeepers	.74	Musicians	.47	Clowns	.22
Kindergarten teachers	.79	Office workers	.51	Crane operators	.19
Makeup Artists	.78	Photographers	.46	Generals	.25
Manicurists	.84	Proof readers	.53	Miners	.16
Nannies	.82	Psychiatrists	.55	Soldiers	.15
Primary school teachers	.75	Supervisors	.45	Toolmakers	.24
Wedding planners	.78	Writers	.50	Wood carvers	.25
Means	.78		.50		.21

In the first step of the questionnaire, the list of occupations was presented without additional adornment on the top of the page, with the space for listing attributes on the bottom of the page. In the second step of the questionnaire, the setup was similar but with the addition of symbols placed next to each occupation. These symbols, unknown to the participants, indicated the gender ratio category that the occupation belonged to. These symbols were ‘✥’ for female-dominated occupations, ‘≎’ for male-dominated occupations, and ▲ for balanced occupations. These were presented in the form ‘[Occupation] [Symbol]’ (e.g., ‘Artists ▲’). The symbols were selected to avoid similarities with common gender-related icons.

#### Procedure

Throughout the survey, participants were instructed that for each occupation they chose, they should as quickly as possible list up to five essential attributes that someone working in that occupation should have. This was done so that attributes were spontaneously created for *all* gender ratio categories. During the experiment, participants were first instructed to select the one profession from the list with which they were the most familiar with, to indicate their level of familiarity with that occupation, and then to list the essential attributes that someone working in that occupation should have. After doing so, they were instructed to select the one profession from the list which they were the *least* familiar with, and were tasked with indicating their level of familiarity with that occupation and listing, as quickly as possible, up to five essential attributes that someone working in that occupation should have. After this, they were instructed to start the second step of the questionnaire.

In the second step of the questionnaire, the symbols were added to the list of occupations. Participants were then instructed to select an occupation from the new list which did not share a symbol with the occupation they had indicated the highest familiarity with in the first step, and to respond in the same way as for the two earlier occupations. Finally, they were instructed to select an occupation that did not share a symbol with either the first or third occupation they had responded to, and were instructed to respond in the same manner as for all previous occupations. As such, at the end of the experiment, participants had responded to three occupations they were familiar with (one from each gender category examined), and one occupation they were unfamiliar with (from any gender ratio category).

#### Results

Due to significant time constraints, data preparation and analysis were undertaken with the primary researcher as the only coder. While this is unusual, it is in keeping with Koivula (2001). All responses (*N* = 479) given by participants were collated into a single document prior to analysis. The results were then examined to determine the number of times each attribute was named. This was done based on the responses being either fully identical or being highly semantically similar (e.g., ‘multitasker’ accepted as ‘ability to multitask’). Initial examination of these 479 responses referred to a total of 230 attributes.

Following initial examination, by-item deselection was undertaken to remove attributes that were occupation-specific (e.g., ‘knows how to sew’; 48 responses – step 1), were specifically gendered (e.g., ‘male’; two responses – step 2), were only named once (105 responses – step 3), or were named by only one participant (twelve responses – step 4). Steps 3 and 4 were in keeping with Glick et al. (1995) and Koivula (2001).

The large number of attributes deselected due to being named only once (*N* = 106) was surprising. Examination of these attributes indicated four major groups; these were *personality traits*^1^ (*N* = 39), *overly broad responses* (*N* = 12) that did not indicate any one attribute in particular (e.g., ‘mundane life’), *overly specific responses* (*N* = 11) that focused on a specific aspect of an attribute (e.g., ‘good reflexes to respond to visual stimuli’), and *physical attributes* (*N* = 7). Four minor patterns of attributes were also identified; these were *negative attributes* (*N* = 5), *occupation-related attributes* (*N* = 5), attributes relating to *mental health* (*N* = 3), and *interpersonal traits* (*N* = 3).

Using the same criteria as were used in the initial examination, the 312 responses left after deselection referred to just 67 unique attributes, with each attribute named in between 2 and 22 responses (*M =* 4.6 responses*, SD* = 3.7 responses). The frequency with which each attribute was named (overall and per gender ratio category), and the number of participants who named each attribute, are presented in Table 2. The results indicated that 20 attributes were named at least once for all three gender ratio categories, while 32 attributes were named at least once for two categories (female-dominated and balanced = 10, female-dominated and male-dominated = 13, male-dominated and balanced = 9), and 15 attributes were named only for one category (female-dominated = 3, balanced = 1, male-dominated = 11).

**Table 2.** Frequency of Naming in Study 1, After Deselection, for Each Attribute Overall and per Gender Ratio Category.

Attribute	Total times named	Female-dominated roles	Balanced roles	Male-dominated roles	# Participants who named attribute
Ability to multitask	4	1	3	0	2
Accuracy	2	0	1	1	2
Acting skills	2	1	0	0	2
Active listening skills	3	1	1	1	2
Adaptable	2	0	1	1	2
Analytical	3	0	3	0	3
Approachable	3	2	0	1	2
Artistic	4	3	0	1	4
Attention to detail	5	1	0	4	4
Attentive	4	2	1	1	4
Authoritative	5	2	1	2	5
Calm	7	2	0	5	6
Careful	3	0	0	3	2
Caring	6	5	1	0	6
Charismatic	3	1	1	1	2
Communication skills	9	6	2	1	5
Confident	7	1	3	3	5
Cooperative	5	1	3	1	5
Creative	21	6	12	3	15
Dedicated	3	1	2	0	2
Determined	3	0	2	1	3
Disciplined	6	0	2	4	5
Effective communicator	3	2	1	0	3
Empathetic	6	4	1	1	5
Fashionable	3	3	0	0	3
Focused	5	1	0	4	5
Friendly	9	3	2	4	8
Funny	4	2	0	2	4
Good sight	2	0	0	2	2
Good with children	3	2	0	1	3
Good work ethic	2	0	0	2	2
Hand-eye coordination	3	0	0	3	3
Hardworking	7	1	2	4	5
Hardy	2	0	0	2	2
Helpful	2	1	0	1	2
Honest	2	0	1	1	2
Imaginative	4	2	2	0	4
Kind	5	5	0	0	5
Knowledgeable	4	2	0	2	3
Leadership skills	9	3	1	5	8
Marketing skills	3	1	1	1	2
Mindful	2	0	0	2	2
Organised	11	7	2	2	8
Passionate	5	1	3	1	4
Patient	22	16	2	4	16
People skills	4	2	2	0	3
Perseverance	3	0	2	1	3
Physical ability	5	0	0	5	5
Practiced	3	1	1	1	2
Precise	4	0	1	3	4
Quick thinking	5	1	0	4	4
Reliable	5	1	2	2	3
Responsible	4	2	2	0	4
Safety focused	2	1	0	1	2
Skilful	3	0	1	2	2
Smart	4	0	3	1	3
Sociable	3	2	1	0	3
Steady handed	2	1	1	0	2
Strategic	2	0	0	2	2
Strong	11	0	0	11	10
Strong presence	2	2	0	0	2
Talented	4	2	2	0	4
Technical skills	6	1	3	2	3
Time management skills	5	2	1	2	4
Tough	2	0	0	2	2
Trustworthy	3	2	0	1	2
Violent	2	0	0	2	2

## Study 2

### Method

#### Participants

This study had 145 participants recruited via a post in a local community group (based in Wellington, New Zealand) on social media. Due to an error in which an explicitly gender marked role (midwives) was accidently included in the initial data collection phase, the results of 13 participants were removed from all analyses. This resulted in the analysis examining the results of 132 participants (87 female, 40 male, 5 non-binary), including 80 university-level students. Participants were between 18 and 53 years old (*M* = 24.33, *SD* = 6.44). All participants were self-reported native New Zealand English speakers. Participants were compensated with an e-voucher equivalent to $10 NZD. This sample size partially matched that of Koivula (2001). Koivula (2001) utilised a between-subjects approach with a total of 403 participants, with each responding to a single role, for a total of 403 data points per attribute. We employed both within-participant and between-participant measures, with 132 participants each responding to three roles, for a total of 396 data points per attribute. Ethical approval for this study was given by the Human Ethics Committee at Victoria University of Wellington.

#### Materials and Research Design

This study was composed of three Likert-scale questionnaires, was presented in English, and was undertaken through the internet-based instrument PsyToolkit (Stoet, 2010, 2017). Participants gave informed consent, answered questions on age, gender, and first language fluency, and stated whether they were currently enrolled university level students prior to starting the study. During the study, participants were presented with one female-dominated, one balanced, and one male-dominated occupation in a semi-random order. These occupations were selected from the same predetermined list used in Study 1, while the attributes shown were the same as were identified in Study 1 after deselection.

Multiple levels of randomisation were used. Firstly, the order in which attributes were shown was randomised by participant and by occupation. Secondly, the order in which the occupations was shown was randomised by participant. Thirdly, the specific occupations an individual was shown was semi-randomised. Specifically, we ensured that a minimum of ten participants responded to each occupational role; this prevented true randomisation. This was done to ensure that all occupations were examined equally, preventing responses to any one specific occupation from biasing the results.

#### Procedure

Over the course of this experiment participants were presented with three occupations. For each occupation, the occupation title was shown at the top of the page, and underneath it was a questionnaire asking students to indicate, as quickly as possible, the importance of each attributes on the list. For each attribute, participants were instructed to indicate the level to which they perceived the attribute as important for an individual working within the occupation to either *be* or to *have*. The Likert scale ranged from 1, *not at all important,* to 7, *vitally important*. The presentation order of these attributes was randomised per participant and per occupation to avoid possible order effects.

#### Data Preparation

The data collected in Study 2 was analysed through linear mixed-effects regression (Analyses 1 & 3) and through factor analysis (Analysis 2). Linear mixed-effects regression provides a measure of participants’ ratings of the *importance* of the attributes (raw data) and components (as result of the component analysis) for the occupations in each gender ratio category. As differences in the task participants undertook may change the salience of components within our social perceptions, we also re-examined the results of Study 1 in light of the components identified in Study 2 through cross-tabulation (Analysis 4). The cross-tabulation of the results obtained in Study 1 provides a measure of the *frequency* with which attributes related to each component was named for each gender ratio category.

Linear mixed-effects regression was used to examine the level to which participants viewed each of the attributes (Analysis 1) or components (Analysis 3) as *important* for each gender ratio category, based on the Likert scale outlined above. During linear mixed effects regression, a process of model fitting is undertaken with all potential experimental and random factors to determine the best possible model (i.e., the ‘model of best fit’) to explain the data. This was done through the *lmer* function of the lme4 package (Version 1.1-18-1; Bates et al., 2015) in R (Version 3.5.1). During this process an initial model was defined, composed of experimental factors (Attribute [Analysis 1] or Component [Analysis 3], Gender Ratio Category [female-dominated vs. balanced vs. male-dominated]), the two-way interaction between Attribute/Component and Gender Ratio Category, and random factors (Participant, Occupation, Occupation Presentation Order, and, for Analysis 3, Attribute). In keeping with Baayen (2008) and Baayen and Milin (2010), refinement to find the model of best fit occurred through back-fitting the fixed effects structure to test whether the experimental factors should be included in the model, forward-fitting the random effects structure to check whether the random factors (including by-participant random slopes of each experimental factor by each random factor) should be included in the model, then re-back-fitting the fixed effects structure to test if the inclusion of the random factors has affected which experimental factors should be included. This was done automatically through the *bfFixefLMER_F*, *ffRanefLMER*, and *fitLMER.fnc* functions of the lme4 package. This provides us with a model of best fit, which can then be examined to determine the most accurate results from the experiment.

Component analysis was undertaken to group attributes into comprehensive factors. Specifically, we used the *fa.parallel* (for parallel analysis) and *principal* (for principal component analysis [PCA] and rotated component matrix [RCM]) functions of the psych package (Version 1.8.10; Revelle, 2017) in R (Version 3.5.1; R Core Team, 2018). As this analysis is exploratory, the parallel analysis was used prior to the PCA to determine the appropriate number of components for the PCA and RCA; *fa.parallel* does this by calculating and comparing the eigenvalues associated with the raw data matrix to those from equally-sized random matrices. Following previous research (e.g., Koivula, 2001) only the attributes and associated Likert ratings were included in these analyses.

The cross tabulation (Analysis 4) allowed for an in-depth examination of frequency data in light of the components identified in Analysis 2. This was important as the way gender information was activated may have differed between the naming and rating tasks. In this analysis the attributes named in Study 1 were assigned labels corresponding to the components identified in Study 2, following which cross tabulation was conducted to examine the frequency with which participants’ spontaneous attribute naming occurred for each components and gender ratio category. This was done through the CrossTable function of the *gmodels* library in R (Version 2.18.1, Warnes et al., 2018).

### Results

#### Analysis 1: Linear Mixed-Effects Regression for Attribute

In this analysis we examined the perceived importance of each attribute for each of the three gender ratio categories. The model of best fit was composed of the experimental factors Attribute and Gender Ratio Category, as well as their interaction, the random factors of Participant, Occupation, and Occupation Presentation Order, and the random slope of Gender Ratio Category by Participant. The results indicated a large and significant main effect of Attribute, *F*(66, 25642) = 137.84, *p < .*001, $\omega_{p}^{2}$ = .239, which was qualified by a medium and significant two-way interaction between Attribute and Gender Ratio Category, *F*(132, 25642) = 49.58, *p < .*001, $\omega_{p}^{2}$ = .080. No significant main effect was found for Gender Ratio Category, *F*(2, 25642) = 9.10, *p* = .112, $\omega_{p}^{2}$ < .001.

The two-way interaction between Attribute and Gender Ratio Category (Table 3) indicated significant differences in the importance of attributes by gender ratio category for 38 of the 67 attributes. For ease of understanding, the results are presented in the text and table in five sections: (a) significantly related to the female-dominated occupations (*N* = 15); (b) significantly related to the male-dominated occupations (*N* = 16); (c) significantly related to the balanced occupations (*N* = 4); (d) significantly different across all gender ratio categories (*N* = 3); (e) no significant differences observed across categories, (*N* = 29). For the first three sections, categorisation was based on the gender ratio category which was the most different from the other two categories, allowing for the identification of negatively-related attributes that are specifically seen as *less* important for one gender ratio category.

**Table 3.** Means and 95% CI for Importance Ratings (7-Point Likert [1,7]) as a Function of Attribute and Gender Ratio Category.

Attribute	Gender ratio category		
	Female-dominated	Balanced	Male-dominated
Significantly related to female-dominated occupations			
Ability to multitask	5.40 [5.02, 5.78]_a_	4.61 [4.22, 5.00]_b_	5.19 [4.80, 5.57]_ab_
Acting skills	2.94 [2.56, 3.32]_a_	2.73 [2.34, 3.12]_ab_	1.97 [1.58, 2.36]_b_
Active listening skills	5.74 [5.36, 6.12]_a_	5.04 [4.65, 5.42]_ab_	4.74 [4.36, 5.13]_b_
Approachable	5.83 [5.44, 6.21]_a_	5.11 [4.72, 5.49]_a_	4.11 [3.73, 4.50]_b_
Calm	5.58 [5.19, 5.96]_a_	4.79 [4.40, 5.17]_b_	5.21 [4.83, 5.60]_ab_
Caring	5.38 [5.00, 5.77]_a_	4.35 [3.95, 4.74]_b_	3.71 [3.32, 4.09]_b_
Determined	4.89 [4.51, 5.27]_a_	5.72 [5.33, 6.11]_b_	5.57 [5.18, 5.95]_ab_
Friendly	5.71 [5.33, 6.09]_a_	4.81 [4.43, 5.20]_b_	4.10 [3.72, 4.49]_b_
Good with children	4.50 [4.11, 4.88]_a_	2.96 [2.57, 3.35]_b_	2.44 [2.05, 2.83]_b_
Helpful	5.69 [5.31, 6.07]_a_	4.78 [4.39, 5.17]_b_	4.50 [4.12, 4.88]_b_
Kind	5.55 [5.17, 5.94]_a_	4.35 [3.96, 4.73]_b_	3.83 [3.44, 4.21]_b_
Patient	5.93 [5.55, 6.31]_a_	5.34 [4.96, 5.73]_ab_	5.15 [4.77, 5.54]_b_
People skills	5.77 [5.38, 6.15]_a_	5.14 [4.76, 5.53]_b_	4.52 [4.14, 4.91]_b_
Responsible	5.83 [5.45, 6.21]_a_	5.04 [4.65, 5.42]_b_	5.55 [5.17, 5.94]_ab_
Sociable	5.43 [5.05, 5.81]_a_	4.50 [4.11, 4.89]_b_	3.82 [3.43, 4.20]_b_
Significantly related to balanced occupations			
Good sight	5.32 [4.94, 5.70]_a_	4.40 [4.01, 4.79]_b_	5.88 [5.50, 6.27]_a_
Hand-eye coordination	5.40 [5.02, 5.79]_a_	4.16 [3.77, 4.55]_b_	6.07 [5.68, 6.45]_a_
Smart	4.23 [3.85, 4.61]_a_	5.04 [4.65, 5.43]_b_	4.35 [3.97, 4.73]_ab_
Steady handed	5.35 [4.97, 5.73]_a_	4.16 [3.77, 4.55]_b_	5.83 [5.44, 6.22]_a_
Significantly related to male-dominated occupations			
Accuracy	5.31 [4.92, 5.69]_a_	5.40 [5.01, 5.79]_ab_	6.09 [5.70, 6.48]_b_
Artistic	5.22 [4.83, 5.60]_a_	4.85 [4.46, 5.25]_a_	3.18 [2.79, 3.57]_b_
Careful	5.71 [5.33, 6.09]_ab_	4.98 [4.59, 5.37]_a_	5.92 [5.53, 6.30]_b_
Charismatic	4.89 [4.51, 5.27]_a_	4.46 [4.07, 4.84]_a_	3.49 [3.10, 3.87]_b_
Creative	5.61 [5.23, 5.99]_a_	5.47 [5.08, 5.86]_a_	3.78 [3.39, 4.17]_b_
Effective communicator	5.66 [5.28, 6.05]_a_	5.71 [5.32, 6.09]_a_	4.85 [4.47, 5.23]_b_
Empathetic	5.19 [4.81, 5.58]_a_	4.61 [4.22, 4.99]_a_	3.61 [3.23, 4.00]_b_
Funny	3.93 [3.55, 4.31]_a_	3.55 [3.16, 3.94]_ab_	2.78 [2.40, 3.17]_b_
Hardy	3.69 [3.31, 4.07]_a_	3.27 [2.89, 3.66]_a_	5.10 [4.72, 5.48]_b_
Imaginative	5.47 [5.08, 5.85]_a_	5.34 [4.96, 5.73]_a_	3.89 [3.50, 4.27]_b_
Marketing skills	4.05 [3.67, 4.43]_a_	4.02 [3.63, 4.41]_a_	2.39 [2.01, 2.78]_b_
Passionate	5.61 [5.23, 6.00]_ab_	5.77 [5.39, 6.16]_a_	4.90 [4.52, 5.29]_b_
Strategic	4.04 [3.66, 4.42]_a_	4.32 [3.93, 4.71]_ab_	4.96 [4.58, 5.34]_b_
Strong	3.19 [2.81, 3.57]_a_	2.83 [2.44, 3.21]_a_	5.51 [5.12, 5.89]_b_
Technical skills	4.38 [4.00, 4.76]_a_	4.82 [4.43, 5.21]_ab_	5.53 [5.14, 5.91]_b_
Tough	3.39 [3.01, 3.77]_a_	2.98 [2.59, 3.37]_a_	5.03 [4.64, 5.41]_b_
Significantly different across all gender ratio categories			
Fashionable	3.89 [3.50, 4.27]_a_	2.89 [2.50, 3.27]_b_	1.69 [1.31, 2.08]_c_
Physical ability	3.97 [3.59, 4.35]_a_	2.78 [2.39, 3.17]_b_	5.89 [5.51, 6.28]_c_
Safety focused	5.32 [4.94, 5.70]_a_	3.85 [3.46, 4.23]_b_	6.26 [5.87, 6.64]_c_
No significant differences observed across categories			
Adaptable	5.51 [5.13, 5.89]_a_	5.47 [5.08, 5.86]_a_	5.50 [5.11, 5.88]_a_
Analytical	3.88 [3.50, 4.26]_a_	4.58 [4.19, 4.97]_a_	4.46 [4.07, 4.84]_a_
Attention to detail	6.21 [5.83, 6.59]_a_	6.05 [5.66, 6.44]_a_	5.88 [5.49, 6.26]_a_
Attentive	5.82 [5.44, 6.20]_a_	5.46 [5.07, 5.84]_a_	5.46 [5.07, 5.84]_a_
Authoritative	4.17 [3.79, 4.55]_a_	3.40 [3.02, 4.35]_a_	3.96 [3.58, 4.35]_a_
Communication skills	5.71 [5.33, 6.09]_a_	5.70 [5.32, 6.09]_a_	4.98 [4.59, 5.36]_a_
Confident	5.27 [4.89, 5.65]_a_	5.19 [4.80, 5.58]_a_	5.33 [4.95, 5.72]_a_
Cooperative	5.37 [4.99, 5.76]_a_	4.97 [4.59, 5.36]_a_	5.02 [4.64, 5.41]_a_
Dedicated	5.39 [5.01, 5.77]_a_	5.73 [5.34, 6.11]_a_	5.61 [5.22, 5.99]_a_
Disciplined	5.14 [4.76, 5.52]_a_	5.19 [4.80, 5.58]_a_	5.73 [5.35, 6.12]_a_
Focused	5.73 [5.35, 6.11]_a_	5.83 [5.44, 6.21]_a_	5.96 [5.58, 6.34]_a_
Good work ethic	5.73 [5.35, 6.12]_a_	5.84 [5.45, 6.22]_a_	6.01 [5.62, 6.39]_a_
Hardworking	5.56 [5.18, 5.94]_a_	5.70 [5.32, 6.09]_a_	6.00 [5.62, 6.38]_a_
Honest	5.23 [4.85, 5.61]_a_	5.00 [4.61, 5.39]_a_	4.87 [4.48, 5.25]_a_
Knowledgeable	5.33 [4.94, 5.71]_a_	5.71 [5.33, 6.10]_a_	5.31 [4.93, 5.70]_a_
Leadership skills	3.95 [3.57, 4.33]_a_	3.83 [3.45, 4.22]_a_	4.00 [3.62, 4.38]_a_
Mindful	5.21 [4.83, 5.59]_a_	4.78 [4.39, 5.17]_a_	4.63 [4.25, 5.01]_a_
Organised	5.74 [5.36, 6.13]_a_	5.53 [5.14, 5.92]_a_	5.24 [4.85, 5.62]_a_
Perseverance	4.91 [4.53, 5.29]_a_	5.54 [5.15, 5.93]_a_	5.58 [5.19, 5.96]_a_
Practiced	5.64 [5.25, 6.02]_a_	5.48 [5.10, 5.87]_a_	6.10 [5.72, 6.49]_a_
Precise	5.74 [5.36, 6.12]_a_	5.27 [4.88, 5.65]_a_	5.91 [5.53, 6.12]_a_
Quick thinking	5.19 [4.81, 5.57]_a_	4.74 [4.35, 5.12]_a_	5.30 [4.91, 5.68]_a_
Reliable	6.04 [5.66, 6.42]_a_	5.36 [4.97, 5.74]_a_	5.79 [5.41, 6.18]_a_
Skilful	5.45 [5.07, 5.84]_a_	5.66 [5.27, 6.05]_a_	5.96 [5.57, 6.34]_a_
Strong presence	4.27 [3.88, 4.65]_a_	4.32 [3.93, 4.71]_a_	4.12 [3.73, 4.51]_a_
Talented	5.05 [4.67, 5.43]_a_	5.51 [5.12, 5.90]_a_	5.00 [4.61, 5.38]_a_
Time management skills	5.71 [5.32, 6.09]_a_	5.42 [5.03, 5.81]_a_	5.35 [4.96, 5.73]_a_
Trustworthy	5.55 [5.16, 5.94]_a_	4.98 [4.59, 5.37]_a_	5.21 [4.82, 5.60]_a_
Violent	1.32 [0.94, 1.70]_a_	1.31 [0.92, 1.70]_a_	1.67 [1.28, 2.06]_a_

*Note.* Per row, means sharing a common subscript (_a_,_b_,_c_) are not significantly [*p* > .5] different.

##### Significantly Related to the Female-Dominated Category

Six attributes (caring, friendly, good with children, helpful, kind, and sociable) were significantly more important for female-dominated occupations than for all other occupations. Five attributes (acting skills, active listening skills, approachable, patient, and people skills) were significantly more important for female than for male-dominated occupations. Three attributes (ability to multitask, calm, and responsible) were significantly more important for female-dominated than for balanced occupations. One attribute (determined) was significantly *less* important for female-dominated than for balanced occupations.

##### Significantly Related to the Balanced Category

One attribute (smart) was significantly more important for balanced than for female-dominated occupations. Three attributes (good sight, hand-eye coordination, and steady handed) were significantly less important for balanced occupations than for all other occupations.

##### Significantly Related to the Male-Dominated Category

Three attributes (hardy, strong, and tough) were significantly more important for male-dominated occupations than for all other occupations. Three attributes (accuracy, strategic, and technical skills) were significantly more important for male than for female-dominated occupations. One attribute (careful) was significantly more important for male-dominated than for balanced occupations. Seven attributes (artistic, charismatic, creative, effective communicator, empathetic, imaginative, and marketing skills) were significantly less important for male-dominated occupations than for all other occupations. One attribute (funny) was significantly less important for male than for female-dominated occupations. One attribute (passionate) was significantly less important for male-dominated than for balanced occupations.

##### Significantly Different Across all Gender Ratio Categories

One attribute (fashionable) was most important for (thus positively related to) female-dominated occupations, and least important for (thus negatively related to) male-dominated occupations. Two attributes (physical ability, safety focused) were most important for (thus positively related to) male-dominated occupations, and least important for (thus negatively related to) balanced occupations.

#### Analysis 2: Component Analysis to Group Attributes into Components

In this analysis we determined the components that each attribute was significantly associated with. The results of the parallel analysis indicated six components. The PCA indicated that these components accounted for a cumulative variance of 53.7%. Examination of competing rotation approaches indicated low inter-component correlations, leading to the selection of Varimax rotation for the RCM.

The results of the RCM (Table 4) indicated the existence of five clear components comprised of 49 of the 67 attributes, freely labelled as Interpersonal Skills (22 attributes; e.g., communication skills, friendly), Precision (9 attributes; e.g., accuracy, hand-eye coordination), Creativity (10 attributes; e.g., acting skills, fashionable), Physicality (5 attributes; e.g., hardy, violent), and Work Identity (4 attributes; e.g., dedicated, hardworking). One component, freely labelled as Intellect, that was only composed of two attributes (Analytical, Smart) and so was discarded (along with the associated attributes), and 15 attributes did not load sufficiently [<0.50] on any of the factors and so were discarded. The remaining five components were then used for further analysis.

**Table 4.** Factor Loadings for Each Attribute per Component From the Rotated Components Analysis.

Attribute	Component					
	1	2	3	4	5	6
Component 1: Interpersonal skills						
Ability to multitask	**.50** ^a^	.18	.08	.18	.26	.14
Active listening skills	**.70** ^a^	.07	.12	.09	.08	.15
Approachable	**.77** ^a^	−0.08	0.13	0.1	−0.16	−0.18
Attentive	**.46** ^b^	.39	−.07	.28	−.14	.09
Authoritative	**.48** ^b^	−.13	.05	.15	.46	.28
Calm	**.60** ^a^	.19	.06	.02	.21	.19
Caring	**.78** ^a^	−.10	.13	.08	−.01	.01
Communication skills	**.64** ^a^	−.13	.12	.17	−.01	.29
Cooperative	**.68** ^a^	.15	−.02	.21	.11	.07
Effective communicator	**.61** ^a^	−.09	.12	.22	−.08	.26
Empathetic	**.71** ^a^	−.08	.13	.27	−.10	−.02
Friendly	**.80** ^a^	−.07	.22	.02	−.07	−.19
Good with children	**.57** ^a^	−.26	.27	−.14	.21	−.16
Helpful	**.77** ^a^	.15	−.06	−.05	.05	.15
Honest	**.65** ^a^	.17	−.05	.07	−.00	.13
Kind	**.80** ^a^	−.09	.22	−.08	.07	−.05
Leadership skills	**.64** ^a^	−.08	−.05	.29	.27	.18
Mindful	**.62** ^a^	.13	.15	.16	.15	.19
Organised	**.51** ^a^	.21	.04	.09	−.03	.40
Patient	**.46** ^b^	.22	.12	.11	.04	.24
People skills	**.75** ^a^	−.11	.26	−.01	.07	−.07
Quick thinking	**.49** ^b^	.05	−.01	.17	.43	.14
Reliable	**.65** ^a^	.28	−.16	−.00	.09	.19
Responsible	**.63** ^a^	.24	−.22	.08	.19	.25
Sociable	**.69** ^a^	−.09	.40	.01	.10	−.19
Time management skills	**.44** ^b^	.23	−.06	.19	−.19	.17
Trustworthy	**.72** ^a^	.17	−.28	.15	−.05	.08
Component 2: Precision						
Accuracy	.03	**.70** ^a^	−.14	.18	−.05	.10
Attention to detail	.05	**.59** ^a^	.09	.07	−.17	.32
Careful	.34	**.58** ^a^	−.14	−.07	.14	.25
Focused	.17	**.45** ^a^	−.01	.37	−.06	.24
Good sight	.08	**.68** ^a^	−.12	−.06	.18	−.05
Hand-eye coordination	−.10	**.69** ^a^	.19	.00	.37	−.28
Practiced	−.01	**.54** ^a^	.15	.20	.11	−.10
Precise	.10	**.73** ^a^	−.05	.04	.03	.23
Skilful	−0.0	**.54** ^a^	.17	.35	.04	−.05
Steady handed	−0.4	**.77** ^a^	.09	.00	.20	−.21
Technical skills	−0.0	**.48** ^b^	.03	.16	.18	.31
Component 3: Creativity						
Acting skills	.22	−.30	**.53** ^a^	.08	.32	−.05
Artistic	−.05	.20	**.80** ^a^	−.08	−.15	−.10
Charismatic	.46	−.20	**.57** ^a^	.10	.14	−.03
Creative	.05	.10	**.81** ^a^	.15	−.16	.01
Fashionable	.14	.13	**.68** ^a^	−.23	.08	−.10
Funny	.41	−.25	**.59** ^a^	−.01	.16	−.04
Imaginative	.11	−.03	**.74** ^a^	.25	−.19	.09
Marketing skills	.08	−.04	**.67** ^a^	−.04	−.05	.22
Passionate	.23	−.06	**.52** ^a^	.46	.02	.05
Talented	−.16	.31	**.58** ^a^	.30	.01	.04
Component 4: Work identity						
Adaptable	.44	.19	−.02	**.47** ^b^	.05	−.16
Confident	.40	−.07	.17	**.41** ^b^	.26	−.06
Dedicated	.20	.12	.21	**.69** ^a^	.12	.07
Determined	.05	.00	.18	**.68** ^a^	.19	.12
Disciplined	.10	.24	−.08	**.41** ^b^	.34	.24
Good work ethic	.36	.22	−.10	**.40** ^b^	.10	.25
Hardworking	.11	.32	−.13	**.63** ^a^	.04	.09
Perseverance	.06	.12	.02	**.69** ^a^	.16	.07
Strategic	.16	.05	.04	**.44** ^b^	.40	.35
Component 5: Physicality						
Hardy	.18	.20	−.02	.23	**.70** ^a^	.08
Physical ability	−.00	.34	−.07	.04	**.72** ^a^	−.09
Safety focused	.41	.28	−.27	−.02	**.46** ^b^	−.05
Strong	.04	.25	−.17	.25	**.71** ^a^	.07
Strong presence	.38	−.22	.26	.29	**.41** ^b^	−.11
Tough	.06	.08	−.13	.23	**.73** ^a^	.15
Violent	−.18	−.12	.16	−.10	**.55** ^a^	−.02
Component 6: Intellect^c^						
Analytical	.24	.09	.06	.15	.27	**.69** ^a^
Knowledgeable	.32	.23	−.00	.39	−.10	**.43** ^b^
Smart	.35	.01	.04	.42	.08	**.55** ^a^

*Note.* Component loadings <.5 are trivial.

^a^These attributes loaded significantly and therefore were included.

^b^These attributes loaded trivially and so were discarded.

^c^This component (and the associated attributes) was discarded as too few attributes loaded significantly upon it.

#### Analysis 3: Linear Mixed-Effects Regression for the Resulting Components

In this analysis we examined the perceived importance of each component for each of the three gender ratio categories The model of best fit was composed of the experimental factors Component and Gender Ratio Category, as well as their interaction, the random factors of Participant, Occupation, Occupation Presentation Order, and Attribute, and the random slope of Component and Gender Ratio Category by Participant. The results indicated small yet significant main effects of Component, *F*(4, 18437) = 10.53, *p < .*001, $\omega_{p}^{2}$ *=* .002, and Gender Ratio Category, *F*(2, 18437) = 4.45 *p* = .012, $\omega_{p}^{2}$ *<* .001, which were qualified by a medium significant two-way interaction between Component and Gender Ratio Category, *F*(8, 18437) = 236.28, *p < .*001, $\omega_{p}^{2}$ *=* .093.

**Figure 1.**
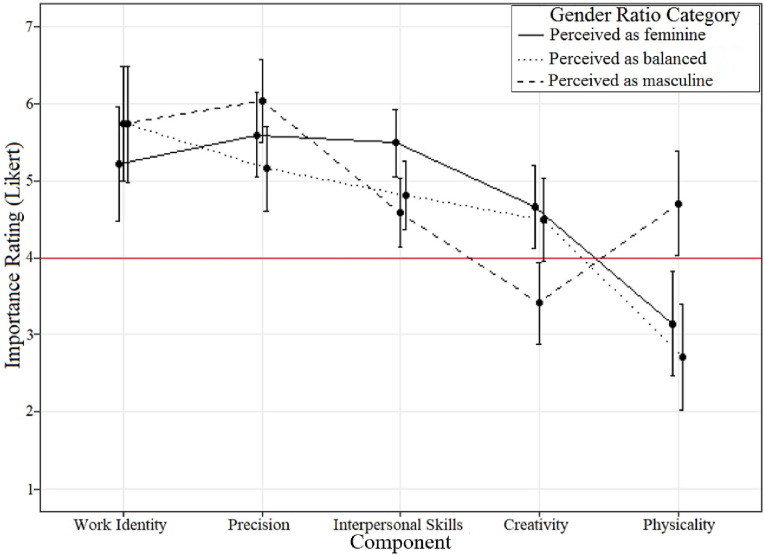
The interaction between Component and Gender Ratio Category in Study 2 (importance ratings). Error bars indicate the 95% confidence interval. The central line (Importance Rating = 4) indicates the point of equilibrium.

The two-way interaction between Attribute and Gender Ratio Category (Figure 1, Table 5) indicated significant differences in perceived importance based on Gender Ratio Category for some, but not all, Components. In the figure, text, and table the results are presented in the order of diminishing importance based on balanced occupations. This was done to allow the balanced occupations to act as a baseline against which the female and male-dominated occupations can be compared. Further, as perceived importance was gathered through a Likert scale, the point of equilibrium (i.e., 4) can be used as a baseline, with responses significantly above 4 indicating that the Component was seen as important for occupations within that Gender Ratio Category, and responses significantly below 4 indicating that the Component was seen as unimportant for occupations within that Gender Ratio Category.

**Table 5.** Means and 95%CI for Importance Ratings (7-Point Likert [1,7]) as a Function of Component and Gender Ratio Category.

Component	Gender ratio category		
	Female-dominated	Balanced	Male-dominated
Work identity	5.22 [4.48, 5.96]_a_	5.74 [4.98, 6.48]_a_	5.74 [5.00, 6.48]_a_
Precision	5.60 [5.05, 6.14]_a_	5.16 [4.60, 5.71]_a_	6.03 [5.49, 6.58]_a_
Interpersonal skills	5.50 [5.05, 5.93]_a_	4.81 [4.36, 5.26]_ab_	4.60 [4.15, 5.03]_b_
Creativity	4.66 [4.13, 5.20]_a_	4.49 [3.95, 5.03]_a_	3.41 [2.88, 3.94]_b_
Physicality	3.14 [2.46, 3.82]_a_	2.71 [2.02, 3.39]_a_	4.70 [4.02, 5.38]_b_

*Note.* Per row, means sharing a common subscript (_a_,_b_,_c_) are not significantly different.

##### Work Identity

Participants viewed Work Identity as being important for all gender ratio categories. There was no significant difference between gender ratio categories. Descriptively, participants viewed Work Identity as less important for female-dominated compared to male-dominated (*M*_Diff_ = 0.52, 95%CI [-0.96, 2.00]) and balanced (*M*_Diff_ = 0.51, 95%CI [-0.98, 2.00]) occupations.

##### Precision

Participants viewed Precision as being important for all gender ratio categories. There was no significant difference between gender ratio categories. Descriptively, participants viewed Precision as important for male-dominated compared to female-dominated (*M*_Diff_ = 0.43, 95%CI [-0.65, 1.53]) and balanced (*M*_Diff_ = 0.87, 95%CI [-0.22, 1.98]) occupations, and more important for female-dominated compared to balanced occupations (*M*_Diff_ = 0.44, 95%CI [-0.66, 1.54]).

##### Interpersonal Skills

Participants viewed Interpersonal Skills as being important for female-dominated occupations, and neither important or unimportant for male-dominated and balanced occupations. Participants viewed Interpersonal Skills as significantly more important for female-dominated compared to male-dominated occupations (*M*_Diff_ = 0.90, 95%CI [ 0.02, 1.78]).

##### Creativity

Participants viewed Creativity as being neither important or unimportant for female-dominated and balanced occupations, yet being unimportant for male-dominated occupations. Participants viewed Creativity as significantly less important for male-dominated compared to female-dominated (*M*_Diff_ = 1.25, 95%CI [0.19, 2.32]) and balanced (*M*_Diff_ = 1.08, 95%CI [0.01, 2.15]) occupations.

##### Physicality

Participants viewed Physicality as being neither important nor unimportant for male-dominated occupations, yet highly unimportant for female-dominated and balanced occupations. Participants viewed Physicality as significantly more important for male-dominated compared to female-dominated (*M*_Diff_ = 1.56, 95%CI [0.20, 2.92]) and balanced (*M*_Diff_ = 1.99, 95%CI [0.63, 3.36]) occupations.

#### Analysis 4: Cross tabulation of the results of Study 1

**Figure 2.**
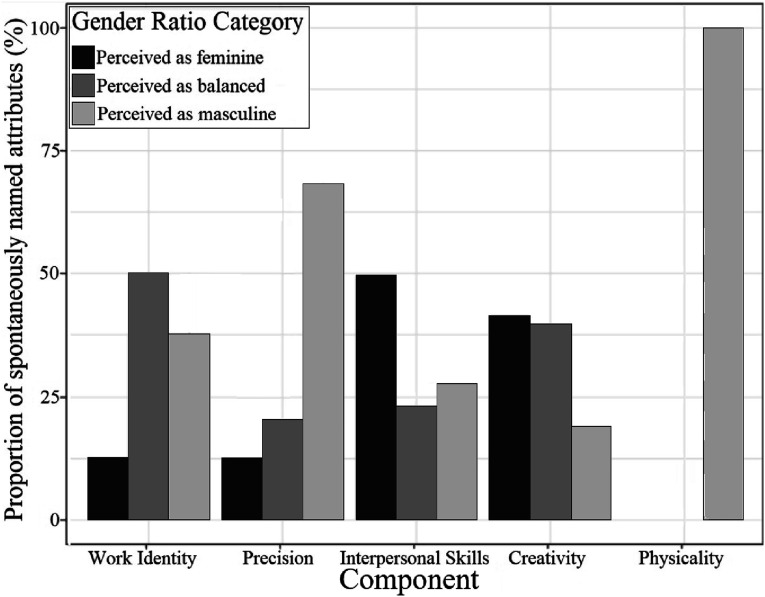
The comparative proportion of spontaneous naming of attributes (Study 1) based on Component and Gender Ratio Category.

In this analysis we examined the rate at which attributes belonging to each component were named (in Study 1) for each of the three gender ratio categories. The results of cross tabulation (Figure 2, Table 6) showed a significant interaction between Component and Gender Ratio Category, Wald *X*^*2*^ (8, *N* = 30) = 70.2, *p < .*001. Table 6 presents frequencies, frequencies as percentages, and each cell’s contribution to the chi-square. As the number of attributes named varied across components, Figure 2 indicates frequencies as percentages for each component. The order of components in Figure 2, text, and Table 6 is in line with the ordering used in Analysis 3.

**Table 6.** Frequency, Frequency as Percentage, and Chi2 Contribution of Spontaneously Named Attributes by Component and Gender Ratio Category.

Component	Gender ratio category	Frequency	Frequency as (, %)	Chi^2^ contribution
Work identity	Female-dominated	2	12.5	2.419
	Balanced	8	50.0	3.440
	Male-dominated	6	37.5	0.001
	*Total*	*16*	*100.0*	*5.860*
Precision	Female-dominated	3	12.0	3.944
	Balanced	5	20.0	0.371
	Male-dominated	17	68.0	5.916
	*Total*	*25*	*100.0*	*10.231*
Interpersonal Skills	Female-dominated	52	49.5	5.575
	Balanced	24	22.9	0.459
	Male-dominated	29	27.6	2.982
	*Total*	*105*	*100.0*	*9.016*
Creativity	Female-dominated	22	41.5	0.492
	Balanced	21	39.6	3.614
	Male-dominated	10	18.9	5.109
	*Total*	*53*	*100*	*9.215*
Physicality	Female-dominated	0	0.0	7.864
	Balanced	0	0.0	5.774
	Male-dominated	22	100.0	22.243
	*Total*	*22*	*100.0*	*35.881*

##### Work Identity

Participants were more likely to spontaneously name attributes when responding about balanced compared to female and male-dominated occupations, and were more likely to spontaneously name attributes when responding about male-dominated compared to female-dominated occupations. This is mostly in keeping with the results of Study 2.

##### Precision

Participants were more likely to spontaneously name attributes when responding about male-dominated compared to female-dominated and balanced occupations, and slightly more likely to spontaneously name attributes when responding about balanced compared to female-dominated occupations. The connection between male-dominated occupations and Precision is in keeping with the results of Study 2, but the large difference in spontaneous naming between male-dominated and female-dominated, and between male-dominated and balanced occupations is not in keeping with Study 2. Further, the pattern for female-dominated and balanced occupations is inverted, again not in keeping with Study 2.

##### Interpersonal Skills

Participants were more likely to spontaneously name attributes when responding about female-dominated compared to male-dominated and balanced occupations, and slightly more likely to spontaneously name attributes when responding about male-dominated compared to balanced occupations. This is mostly in keeping with the results of Study 2, although the pattern for the male-dominated and the balanced occupations is inverted.

##### Creativity

Participants were more likely to spontaneously name attributes when responding about female-dominated and balanced occupations compared to male-dominated occupations, and were very slightly more likely to spontaneously name attributes when responding about female-dominated compared to balanced occupations. This is fully in keeping with the results of Study 2.

##### Physicality

Participants only spontaneously named attributes connected to this component for male-dominated occupations. This is mostly in keeping with the results of Study 2.

## General Discussion

This paper explores how using gender ratio information to select stimuli affects the activation of gendered social information, and explores whether such stimuli activate gender stereotype knowledge and/or conceptual gender knowledge. The study is an exploratory examination of these effects. We conducted two studies to examine gender ratio information at both the individual attribute level and the ‘grouped attributes’ component level using attribute naming (Study 1) and rating (Study 2). As this was an exploratory study, no hypotheses were formulated regarding the nature of gender ratio information itself. The results indicated that distinctions between gender categories were found for some, but not all, attributes/components, indicating support for both the conceptualisation of gender ratios as measuring gender stereotypicality (clear distinctions) and categorical gender (no distinctions), *if* researchers are willing to accept this overlap.

At the attribute level, the results of the naming task indicated that attributes were slightly more likely to be named in relation to occupations throughout all three categories (*N* = 20) than in just one category (*N* = 15), but that most attributes were named in relation to two categories (*N* = 32). Further, for the rating task, most attributes showed significant differences in importance between at least two gender ratio categories (*N* = 38), with the 29 attributes that showing no significant differences in importance between gender ratio categories. These findings offer support for both conceptualisations, as well as their overlap, with some preference for categorical gender in attribute naming and gender stereotypicality in attribute rating.

At the component level, the results of Study 2 indicated that three of the five components (*creativity*, *interpersonal skills*, and *physicality*) showed significant differences in importance between at least two gender ratio categories while two components (*precision* and *work identity*) showed no significant differences between gender ratio categories. The three components that showed significant differences between gender ratio categories are also in line with gender stereotype categories identified in research outside of gender ratio information (e.g., Cejka & Eagly, 1999). The results of Study 1 largely supported these findings aside for *precision*, which was named far more commonly for male-dominated occupations than for female-dominated and balanced occupations.

Taken together, the results suggest that the beliefs associated with the occupations selected based on their gender ratio are not at the ‘logical extreme’ for either gender stereotypicality (i.e., clear, significant, distinctions between all gender ratio categories) or conceptual gender (i.e., no significant distinctions between any gender ratio categories). A possible explanation for this is that gender information, when cognitively activated, provides a measure by which gender-based categorisation can occur, with some of this information remaining highly salient after categorisation (i.e., those with clear separations between gender ratio categories) and some losing salience after categorisation (i.e., those with clear overlaps between gender ratio categories).

To summarise, gender ratios can be used to guide stimulus selection for research into both gender stereotypicality and conceptual gender for researchers who are willing to accept that some, but not all, gender ratio information remains salient after categorisation. As such, gender ratios should not be used to guide stimulus selection for research that requires either that activated gender information remains fully salient (gender stereotypicality), or that it loses all salience (conceptual gender).

The exact way in which components differed by gender ratio category is also worth discussing, with the results suggesting both positive/prescriptive gendered beliefs and negative/proscriptive gendered beliefs. In relation to positive/prescriptive beliefs, one component was perceived as specifically important for female-dominated occupational roles (*interpersonal skills*; significantly more important for female compared to male-dominated occupations) and two components were perceived as specifically important for male-dominated occupational roles (*physicality* and *precision*; perceived as significantly [physicality] and descriptively[precision] more important for male-dominated compared to balanced and female-dominated occupations). In relation to negative/proscriptive beliefs, one component were perceived as specifically not important for female-dominated occupational roles (*work identity*; descriptively less important for female-dominated compared to balanced and male-dominated occupations), and one component was seen as specifically not important for male-dominated occupational roles (*creativity*; significantly less important for male-dominated compared to balanced and female-dominated occupations). These findings suggest that gendered information associated with role nouns informs gender categorisation by leading the observer to accept (positive/prescriptive beliefs) or reject (negative/proscriptive beliefs) specific gender categories. This pattern was also found at the attribute level, indicating that positive/prescriptive and negative/proscriptive beliefs affect social perception at both the macro (component) and micro (attribute) level. These results hold important implications for future research – as gender stereotypicality has been found to affect occupation selection (e.g., Eccles, 2011), these findings suggest that there is at least a subset of female-dominated (and male-dominated) roles that have a specific ratio due to being negatively/proscriptively associated with men (or women). Indeed, this may explain some of the variability found across studies that have used gender ratios to guide stimulus selection, as experimental noise may have been added due to negative/proscriptive beliefs associated with one gender being misattributed as general beliefs associated with another gender (e.g., a role seen as specifically inappropriate for men [but equally appropriate for women and mixed gender groups] being coded as a feminine-stereotyped group). As such, future research should seek to determine whether the gender ratio associated with different roles is due to positive/prescriptive or negative/proscriptive beliefs, with these findings then allowing for more nuanced examinations of gender stereotypicality and conceptual gender (among others). These findings, and future research along these lines, are important not only in academia; for example, gender equality initiatives aimed at reducing horizontal gender segregation may benefit from knowing whether interventions within a particular industry should focus on overcoming prescriptive or proscriptive beliefs about individuals’ suitability for that industry.

It is important to note that while Creativity was found to be negatively/proscriptively associated with male-dominated roles in both component level analyses (Analyses 3 and 4), Work Identity was only found to be negatively/proscriptively associated with female-dominated roles in our frequency analysis (Analysis 4). This may suggest that negative/proscriptive beliefs associated with male-dominated roles may generate richer and more specific representations than negative/proscriptive beliefs associated with female-dominated roles. This would be consistent with previous research (e.g., Koivula, 2001). Our results at the attribute level support this interpretation, as more attributes were spontaneously named for male-dominated occupations than for female-dominated occupations (11 male-dominated vs. 3 female-dominated). Further, during the rating task, more attributes were significantly *negatively* related to male compared to female-dominated occupations (10 male-dominated vs. 1 female-dominated), while more attributes were significantly *positively* related to female compared to male-dominated occupations (13 female-dominated vs. 7 male-dominated). These results suggest that, at the attribute level, ideas of femininity are based more on what individuals *should* do (prescriptive beliefs), whereas ideas of masculinity are based more on what individuals *should not* do (proscriptive beliefs).

The studies in this paper have a number of potential limitations. Firstly, the English norms provided by Misersky et al. (2014) were obtained from participants in the United Kingdom. Therefore, it is possible that an identical experiment conducted with participants from the United Kingdom might yield slightly different results. However, gender ratios have been found to be relatively stable across languages and cultures, with female-dominated and male-dominated roles tending to be so regardless of language (e.g., Gabriel et al., 2023; Misersky et al., 2014). Further, cross-cultural monolingual research that has used gender ratios as a basis for stimulus selection has found that L1 speakers of the same language in cultures separated by large geographical distances produce very similar results (e.g., Kim et al., 2023). As the female- and male-dominated roles used in this paper were selected to be as highly gender-dominated as possible, we believe that the differences in the results obtained in New Zealand and those that could be obtained in the United Kingdom would be relatively small. However, further research is needed to determine whether this is the case.

Secondly, although the symbols used in Study 1 were selected to avoid reminding participants of gender-related symbols, it is possible that participants, in choosing which of the role sets associated with a particular symbol to select, might have noticed the gender information associated with that role set. If this is the case, we believe that this would have led to higher response noise, as some participants would have named far more stereotypically/typically gendered attributes than they would otherwise, and some participants would name far more counter-stereotypically/counter-typically gendered attributes than they would otherwise. As participants could only name a certain number of attributes per role, it is possible that this increased noise prevented some attributes from being named sufficiently often to be included in Study 2 and the data analysis. Future research following this paradigm might then benefit from computer-mediated surveying, as each role that participants respond to could be used to refine the roles presented in the next step (e.g., if participants initially responded to a female-dominated role, in the next step they would be asked to choose from a list of male-dominated and balanced roles).

Lastly, although our study assumes that all respondents were native speakers of New Zealand English, we cannot be sure that this was the case for all participants. More specifically, because Study 2 was internet-based, we cannot be confident that all demographic information provided by participants was correct (e.g., Kim, 2023; Reips et al., 2015). If L2 English speakers took part, it could have resulted in more experimental noise. However, the alternative – conducting data collection in the laboratory – might still yield slightly different results, as responses given at home (i.e., in an environment familiar to the participant) have a higher level of ecological validity (e.g., Kim, 2023; Reips et al., 2015).

In conclusion, the study of social perceptions of roles selected based on their perceived gender ratio revealed five components and their associated attributes. The results of the attribute naming and rating tasks were examined using linear mixed-effects regression, component analysis, and frequency analysis. The results help to provide a richer image of the nature of gender ratio information, and support the use of gender ratio as a measure of both gender stereotypicality and conceptual gender if researchers are willing to accept that attributes/components sometimes remain salient (against conceptual gender) and sometimes lose salience (against gender stereotypicality) following classification, but do not support it as a measure of either if it is required to be unambiguous. Crucially, our results suggest the existence of negative/proscriptive gendered beliefs that guide the perception of particular roles at both the attribute and component level, which has important implications for future research.

## Data Availability

Due to the terms outlined by the Human Ethics Committee, data from Study 1 (attribute naming task) cannot be made available due to the potential identifiability of participants. Data from Study 2 that has been appropriately anonymized, along with analytical scripts, is available from NTNU Open Data (https://dataverse.no/dataverse/ntnu). *

## Note

1. It is interesting to note that personality traits were the most common attributes to be named only once. It is possible that there is a far greater number of unique descriptors for personality traits than for physical, occupational, and interpersonal attributes. For example, a worker displaying the interpersonal trait of ‘strong communication skills’ when talking to a customer might be perceived as displaying the personality traits of cheerfulness, friendliness, charm, or being bubbly.
